# Genetic diversity of BCoV in Brazilian cattle herds

**DOI:** 10.1002/vms3.102

**Published:** 2018-04-24

**Authors:** Adeline de Mira Fernandes, Paulo E. Brandão, Michele dos Santos Lima, Maira de Souza Nunes Martins, Thais G. da Silva, Vivian da Silva Cardoso Pinto, Larissa T. de Paula, Marta Elisabete S. Vicente, Liria H. Okuda, Edviges M. Pituco

**Affiliations:** ^1^ Laboratory of Bovine Viruses Center of Research and Development of Animal Health Biological Institute of São Paulo São Paulo Brazil; ^2^ Department of Preventative Veterinary Medicine and Animal Health Faculty of Veterinary and Zootechnical Medicine University of São Paulo São Paulo Brazil

**Keywords:** bovine coronavirus, genealogy, gene N, sequencing

## Abstract

Bovine coronavirus (BCoV) is one of the main aetiological agents of gastroenteritis in calves, causing significant economic damage to livestock. This study aims to characterise BCoV genetically on the basis of the N gene. A total of 114 faecal samples from beef and dairy calves with or without clinical symptoms of diarrhoea from five Brazilian states (São Paulo, Minas Gerais, Santa Catarina, Mato Grosso and Bahia) were evaluated between 2008 and 2015 by technique of Semi‐*nested* RT‐PCR for gene N and genealogical analysis. Of the 114 samples analysed, 14.91% (17/114) were positive. BCoV was detected in 22.72% (10/44) of the animals with diarrhoea and in 10% (7/70) of asymptomatic animals. BCoV was identified in calves from rural properties located in all of the regions sampled. Genealogical analysis showed that the Brazilian sequences of BCoV for the gene which codes for the N protein can be broken down into two distinct clusters, and the samples from this study were closely linked to Asian strains. These results contribute to the molecular characterization of BCoV in Brazil and are the first report of the circulation of BCoV in the states of Santa Catarina and Bahia.

## Introduction

BCoV belongs to the order *Nidovirales*, family *Coronaviridae*, sub‐family *Coronavirinae* within the genus *Betacoronavirus*. BCoV consists of a non‐segmented, single‐stranded, positive‐sense RNA (ssRNA) (Wentworth & Holmes 2007).

Structurally, it is an enveloped virus with a diameter of approximately 100–120 nm with five structural proteins identified as: (S) spike glycoprotein; (M) integral membrane protein; (HE) hemagglutinin‐esterase glycoprotein; (E) small membrane protein and (N) nucleocapsid phosphoprotein (Wentworth & Holmes 2007; Asadi *et al*. 2015).

The N protein is a phosphoprotein, rich in basic amino acids and directly linked to the genomic RNA, forming a helicoidal nucleocapsid. The N protein carries out various functions linked to viral pathogenesis, transcription and replication. It is often used for molecular diagnosis of BCoV as it is a highly conserved protein that is expressed in large quantities during viral replication (Saif 2010).

BCoV is considered to be one of the main aetiological agents of neonatal diarrhoea in calves, causing significant economic damage to dairy and beef livestock. It is also linked to winter dysentery in adult cows and to respiratory diseases (Asadi *et al*. 2015).

Infections with BCoV are endemic and can spread around the world. Their frequency varies substantially, primarily due to geographical distribution, breeding and farming systems (Bolieau & Kapil 2010; Asadi *et al*. 2015).

Treatment for BCoV infections is non‐specific. Administration of antibiotics may prevent secondary or opportunistic infections. The fluid therapy is used as a primary supportive therapy in cases of gastroenteritis. Vaccination is recognized as an effective prophylactic measure (Heckert *et al*., 1991; Saif 2010). Live or inactivated vaccines in combination with Rotavirus, *Escherichia coli* and *Clostridium perfringens* (Kapil *et al*., 1999) are commercially available on the market. However, in Brazil, there is no national BCoV vaccination programme and information on the immunization of the herds is non‐existent.

Despite its significance for cattle breeding, there have been few and limited systematic and comprehensive studies of the occurrence and molecular characterisation of BCoV in Brazil, which prevents full understanding of the epidemiology of the disease. Comparisons between strains of BCoV have been mainly based on the partial or full sequencing of the genes which code for S and HE proteins, but there is only a small amount of information relating to the N gene. The objective of this study is therefore to genetically characterise BCoV on the basis of the partial sequencing of the gene which codes for the N protein in faecal samples from calves with and/or without clinical symptoms of diarrhoea.

## Material and methods

### Reference virus

The Kakegawa (Akashi *et al*. 1980) strain of coronavirus which propagates in HmLu‐1 (hamster lung) cells with a hemagglutination titre of 256 and DEPC‐treated water was used as positive and negative controls respectively.

### Sampling

This study was conducted with appropriate faecal samples received from the Laboratory of Bovine Viruses at the Biological Institute of São Paulo, Brazil, during the period from 2008 to 2015. The samples were collected using swabs or directly from the rectum and stored at −20°C.

A total of 114 faecal samples was collected from calves, with or without clinical symptoms of diarrhoea, both sexes, aged between 1 and 15 months from 15 dairy and beef herds from 12 municipalities in the southern (Santa Catarina), south‐eastern (São Paulo and Minas Gerais), central‐western (Mato Grosso) and north‐eastern (Bahia) regions of Brazil (Table 1). The animals had not been vaccinated against BCoV. The faecal samples were prepared as 20% suspensions in 0.01 mol/L phosphate‐buffered saline (PBS)/BSA at 0.1% pH 7.2. The swabs contained in the MEM medium and the faecal samples were homogenized and clarified at 12 000xg/30 min and the supernatant stored at −20°C until the analysis was carried out.

**Table 1:** Samples analyzed by location, age, sex, exploration and animal breeding

Locality	Samples/Herd	Sex	*n*
**São Paulo**	33/7	Male	46
Angatuba	1/1	Female	36
Bragança Paulista	1/1	NI^a^	32
Cotia	2/1	**Animal brealing**	*n*
Eldorado	2/2	Dairy cutting	40
Pindamouhangaba	25/1	Dairy fanning	33
Descalvado	2/2	NI	41
**Minas Gerais**	16/2	**Exploration**	*n*
Alpinópolis	8/1	Extensive	21
Silvanópolis	8/1	Intensive	25
**Santa Catarina**	1/1	Semi‐intensive	3
Jaguaruna	1/1	NI^a^	65
**Bahia**	44/2	**Age**	*n*
Campo Alegre	11/l	= 1 month	8
ltamarajú	33/l	2‐3 months	27
:**Mato Grosso**	20/l	4–5 months	5
lpiranga do Norte	20/1	>6 months	36
		NI^a^	38

a NI, Not Identified

### Semi‐*nested* RT‐PCR for gene N

Viral RNA was extracted, using the TRIzol method (Invitrogen™) in accordance with the manufacturer's specifications and store at −80°C until analysis. In order to detect BCoV, a reaction was carried out with semi‐nested RT‐PCR using specific primers for the gene that codes for BCoV N protein, as described by Asano *et al*. (2009). The RT‐PCR reaction was carried out by adding 2.5 *μ*L of RNA to the PCR mix (25 *μ*L of 2x Reaction Mix (Invitrogen™), 0.2 mmol/L of BCoV1 (5′AGAGCTCAAYCCAAGCAA ACTGY 3′) and BCoV2 (5′AG CAGACCTTCCTGAGCCTTCAAT 3′), 2 *μ*L of SuperScript III RT Mix /Platinum Taq (Invitrogen™) and 16 *μ*L of DEPC‐treated water to achieve a final volume of 25 *μ*L, heated to 94°C/4 min followed by 35 cycles of 94°C/1 min, 50.2°C/30 s, 72°C/45 s and 72°C/5 min for the final extension.

The second amplification was performed by adding 2.5 *μ*L of the product from the first amplification to the PCR *mix* containing 25 *μ*L of PCR Master Mix (50U/mL Taq polymerase, 400 *μ*mol/L for each dNTP, and 3 mmol/L MgCl2), 0.2 mmol/L de BCoV1 (5′AGAGCTCAAYCCAAGCAAACTGY 3′) and BCoV3 (5′TCAATRTCGGTGCC ATACTGGTCT 3′) and 18 *μ*L of DEPC‐treated water to achieve a final volume of 25 *μ*L and heated to 94°C/4 min followed by 35 cycles of 94°C/30 s, 50.2°C/30 s, 72°C/45 s and 72/5 min for the final extension. Two microliters of the product from the semi‐nested RT‐PCR were analysed in 1.5% agarose gel stained with GelRed™ (1:150) and observed under ultraviolet light. The samples which showed *amplicons* of 306pb in length were considered positive.

### DNA Sequencing and Genealogical Analysis

The fragments of 306 pb corresponding to gene N were purified using the QIAquick PCR purification kit (Qiagen), visually quantified using GeneRuler^®^ 100 bp DNA,sequencer DNA 3500XL GeneticAnalyzer (AppliedByosystems^®^) with the BigDye^®^Terminator v3.1 (AppliedByosystems^®^) in accordance with manufacturer's instructions.

The chromatograms generated for each of the sense and antisense sequences of each sample were subjected to the application Phredonline on http://asparagin.cenargen.embrapa.br/phph/ to evaluate their quality. The final sequence for each sample was obtained, using the application Cap‐Contig with a Bioedit program v.5.0.9 (Hall 1999). This was then submitted to BLAST/n to for sequencing confirmation on http://www.ncbi.nlm.nih.gov/BLAST. The final sequences for each sample^1^ were aligned with the analogous sequences of BCoV retrieved from the *GenBank* (Appendix S1), using the Bioedit program. The identities of the sequences of nucleotides aligned with a CLUSTAL/W matrix were calculated using the Bioedit program. The alignments were used to generate genealogical trees using the maximum likelihood, heuristic method Nearest‐Neighbor‐Interchange, plus gamma distribution and invariant sites and Jukes‐Cantor algorithm with 1000 *bootstrap* iterations using the MEGA 6 program.

### Data analysis

Microsoft^®^ Office Excel 2010 was used for identity mean and standard deviation calculations.

## Results

### Detection of BCoV in faecal samples from calves

Of a total of 114 faecal samples analysed by means of semi‐*nested* RT‐PCR targeting gene N, 17 (14.91%) were positive for BCoV. Of the animals that were positive for BCoV, 22.72% (10/44) of the calves had diarrhoea and 10% (7/70) were asymptomatic.

In terms of the geographical distribution, BCoV was found in the states of São Paulo, Minas Gerais, Santa Catarina, Mato Grosso and Bahia. The percentages of positive results for coronavirus by state were: Bahia [7.01%(8/114)]; São Paulo [2.6%(3/114)]; Mato Grosso [2.6%(3/114)]; Minas Gerais [1.7%(2/114)]; and Santa Catarina [0.8%(1/114)].

### Sequencing and genealogical analysis

Viable nucleotide sequences for the gene N were obtained (Phred score ≥ 20) in 8 of the 17 samples which were positive for BCoV. Sequence analyses for the gene N using BLAST/n confirmed the identity of these, and no non‐analogous sequences were found.

Taking into account identity mean and standard deviation for nucleotides, the percentage of overlap between the identity of the nucleotides in the samples from this study and Brazilian sequences was 88.6% ± 0.006 SD. It was also possible to observe a large number of nucleotide identities (97.0% ± 0.012 SD) with Asian‐derived sequences. In terms of the samples which originated from Europe and North America, the identities were 96.1% ± 0.016 SD and 96.6% ± 0.09 SD respectively.

In the comparison with BCoV Mebus prototype, evidence was found of nucleotide substitutions exclusively in field samples sequenced in five positions, corresponding to the changes in amino acids. Position 9849 of the amino acid sequence of the gene N showed the substitution of a leucine by a histidine in all field samples.

For sample 2188/15, synonymous mutations occurred in position 9893, and non‐synonymous mutations in positions 9850 and 9917, with the substitution of leucine by arginine and asparagine by serine respectively. No insertions or deletions were observed.

**Figure 1:**
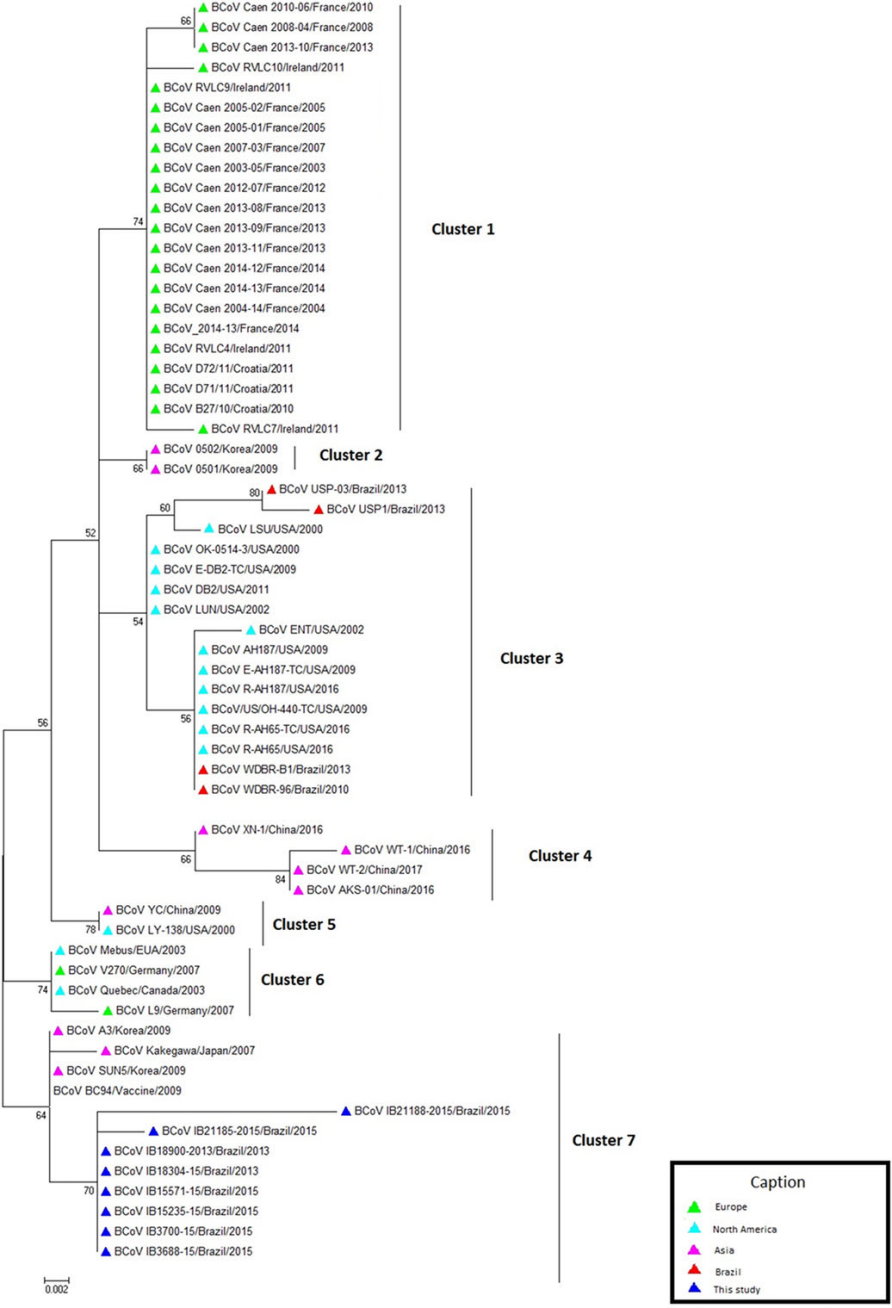
Non‐rooted phylogenetic tree constructed based on protein sequence maximum likelihood criteria and the Jukes‐Cantor evolutionary model for nucleotide sequences of the gene which codes for N protein. The phylogenetic tree is denominated by: Sequence name/Country/year.

The tree of maximum likelihood for nucleotide sequences for 8 samples of BCoV resulted in seven paraphyletic and polyphyletic *clusters* for the gene N (Figure 1).The *Cluster* 1 consisted of European strains from France (CAEN and 2014‐13), Ireland (RVLC4, RVLC9 and RVCL10) and Croatia (D71/11, D72/11 and B27/10) respectively. *Cluster 2* was composed exclusively by two Korean sequences of BCoV (0501 and 0502). *Cluster 3* consisted Brazilian samples (USP1, USP 03, WDBR‐B1 and WDBR‐96) along side North American strains (LSU, OK 0514‐3, E‐DB2‐TC, DB2, LUN, ENT, AH187, EH187, RH187, US/OH440, RAH65 and RAH65‐TC). *Cluster 4* was formed by sequences of BCoV from China (XN‐1, WT‐1, WT‐2 and AKS‐01). *Cluster*s 5 and 6 consisted of samples originating from USA and Canada (LY‐138, MEBUS and QUEBEC), Germany (V270 and L9) and China (YC). Samples in this study in *Cluster* 7 were Asian strains (A3, SNU5 and Kakegawa) originating from Korea and Japan and vaccine strains (BC94). In terms of host age, BCoV sequences from calves and adult cows were found together in field samples in this study, in addition to others retrieved from *GenBank*.

## Discussion and conclusion

Diarrhoea is a worldwide morbid condition and BCoV is one of the main causes of gastroenteritis in young animals (Bolieau & Kapil 2010; Asadi *et al*. 2015).

Infections with BCoV have been described in different geographical regions including Brazil (Brandão *et al*. 2002; Jerez *et al*. 2002; Barry *et al*.^2009^; Stipp *et al*.^2009^; Barros *et al*. 2013; Lorenzetti *et al*. 2013; Coura *et al*. 2015). The results of this study show the occurrence of BCoV in the states of São Paulo, Minas Gerais, Bahia, Mato Grosso and Santa Catarina, which indicates the dissemination of the agent in cattle herds in Brazil. This study is the first report of the circulation of BCoV in the states of Santa Catarina and Bahia, regions in the south and north‐east of the country.

BCoV was detected both in calves with clinical symptoms of diarrhoea and asymptomatic calves. The presence of coronavirus in asymptomatic calves, or carriers, indicates that these animals can act as a source of infection for animals which are susceptible to the disease (Jerez *et al*. 2002; Stipp *et al*. 2009).

On the basis of analyses of the partial sequence of gene N nucleotides, a high degree of identity was confirmed between the samples sequenced in this study and Asian samples of BCoV. Low identity percentages correspond to Brazilian strains, demonstrating a great variability in relation to the N gene in Brazilian BCoV sequences.

Although the N protein is conserved as it has a lower propensity for polymorphism than proteins S and HE, synonymous point mutations and non‐synonymous mutations can also occur. Since the Brazilian BCoV sequences were detected in different locations and periods, these results may be linked to factors such as geographical and temporal distribution, different breeds and breeding systems and animal marketing, thus exerting a positive selective pressure for BCoV N gene.

The tree of maximum likelihood for the N gene (Figure 1) shows that sequenced field samples diverged from other sequences of BCoV which have already been described in Brazil, with evidence being found of the existence of two different *clusters*. The Brazilian samples analysed here can be differentiated from Asian strains originating in Japan and Korea.

On the basis of these data, it is initially possible to infer that at a certain moment in evolution, nucleotide substitutions have given rise to two or more genotypes of BCoV which are spreading to several countries. This is compatible with some previous descriptions (Barros *et al*. 2013; Bidokhiti *et al*. 2013; Bok *et al*. 2015; Gunn *et al*. 2015; Lojkic *et al*. 2015). Another plausible hypothesis for the convergence of the sequences derived from countries that are so far apart is the fact that the majority of the strains of BCoV contained in this *cluster* were isolated in cell cultures before being sequenced. Isolation in cell culture before sequencing exerts artificial selection, inducing convergent development between different samples (Borucki *et al*. 2013).

In terms of the age of the host, the strains of BCoV in calves were the same as in adult animals regardless of geographical location, showing a significant identity between BCoV associated with neonatal diarrhoea and winter dysentery, which explains the rapid dissemination of BCoV within a herd before mutations have accumulated.

The genealogical analysis of the partial sequencing of the gene that codes for N protein shows that Brazilian samples of BCoV can be broken down into two distinct *clusters* (Figure 1), with the samples in this study being strictly linked to Asian strains.

The genetic variability of BCoV has a direct implication for methods of diagnosis based on molecular biology and on the production of vaccines. It is also emphasized that in addition to genetic monitoring, evaluation of immunization efficacy and an effective vaccination programme are crucial for the control of BCoV infections.

These results emphasise the importance of molecular characterisation of BCoV, enabling a more detailed understanding of the dynamics and pattern of development – elements which are fundamental when planning epidemiological action.

## Source of funding

This study did not receive funding from public or private sector agencies.

## Conflicts of interest

The authors declare no conflicts of interest in relation to this work.

## Ethics statement

This study was approved by the Ethics Committee for Experimentation on Animals of Biological Institute of São Paulo and conducted in accordance with national and international guidelines on handling animals.

## Contributions

Design of the experiments: Adeline de Mira Fernandes and Michele dos Santos Lima. Samples collection: Adeline de Mira Fernandes, Paulo Eduardo Brandão and Liria Hiromi Okuda. Experiments: Adeline de Mira Fernandes, Marta Elisabete Scarelli Vicente and Maira de Souza Nunes Martins. Genealogical analysis: Paulo Eduardo Brandão. Manuscript draft and Revison: Adeline de Mira Fernandes, Paulo Eduardo Brandão, Michele dos Santos Lima, Maira de Souza Nunes Martins, Thais Garcia da Silva, Vivian da Silva Cardoso Pinto, Larissa Tuffani de Paula, Liria Hiromi Okuda and Edviges Maristela Pituco.

## Supporting information


**Appendix S1.** BCoV sequences based on the gene encoding the N protein recovered from GenBank, according to the accession number, identification, country of origin, type of sample and year of sequencing.

## Note

1 GenBank accession numbers for the N gene sequences of this study(21188/15, 21185/15, 18900/13, 18304/15, 15571/14,15235/15,3700/15 and 3688/15) are : MF510415, MF510416, MF510417, MF510418, MF510419, MF510420, MF510421 and MF510422, respectively.
