# Supplementary material for: Genetic diversity of BCoV in Brazilian cattle herds
Source: Vet Med Sci. 2018 Apr 24;4(3):183–9. doi: 10.1002/vms3.102 (PMC6090412; doi:10.1002/vms3.102)
Supplement: Supplementary file 1 — Appendix S1. BCoV sequences based on the gene encoding the N protein recovered from GenBank, according to the accession number, identification, country of origin, type of sample and year of sequencing. [file VMS3-4-183-s001.docx]

| Acess  number | Strain | Strain origin | Contry | Year |
| --- | --- | --- | --- | --- |
| EU401985 | BC94 | Vaccine | Korea | 2009 |
| EU401984 | SUN5 | Cell | Korea | 2009 |
| EU401983 | A3 | Cell | Korea | 2009 |
| EU401981 | 0502 | Faecal | Korea | 2009 |
| EU401980 | 0501 | Faecal | Korea | 2009 |
| KX266233 | WT2 | Faecal | China | 2017 |
| KX073970 | XN-1 | mesentery lymph node | China | 2016 |
| KU886219 | AKS-1 | NI*  NI* | China | 2016 |
| FJ556872 | YC | NI* | China | 2009 |
| AB354579 | Kakegawa | Cell | Japan | 2007 |
| EF193074 | V270 | Faecal | Germany | 2007 |
| EF193073 | L9 | Cell | Germany | 2007 |
| U007352 | Mebus | Cell | USA | 2003 |
| AF058942 | LY-138 | NI* | USA | 2000 |
| DQ811784 | DB2 | NI* | USA | 2011 |
| FJ938065 | AH187 | Nasal | USA | 2009 |
| FJ938064 | E-AH187 | Cell | USA | 2009 |
| EF424620 | R-AH187 | Cell | USA | 2016 |
| AF391541 | ENT | Cell | USA | 2002 |
| AF05 8944 | OK-0514-3 | Cell | USA | 2000 |
| FJ938063 | EDB2-TC | NI* | USA | 2009 |
| FJ938066 | US/OH-440 | - NI* | USA | 2009 |
| EF424618 | R-AH65-TC | Cell | USA | 2016 |
| AF391541 | R-AH65 | Nasal | USA | 2016 |
| AF058943 | LSU | Cell | USA | 2000 |
| AF220295 | Quebec | Cell | Canada | 2003 |
| KT318096 | Caen 2004-14 | Faecal | France | 2004 |
| KT318095 | Caen 2014-13 | Faecal | France | 2014 |
| KT318094 | Caen 2014-12 | Faecal | France | 2014 |
| KT318093 | Caen 2013-11 | Faecal | France | 2013 |
| KT318092 | Caen 2013-10 | Faecal | France | 2013 |
| KT318091 | Caen 2013-09 | Faecal | France | 2013 |
| KT318090 | Caen 2013-08 | Faecal | France | 2013 |
| KT318089 | Caen 2012-07 | Faecal | France | 2012 |
| KT318088 | Caen 2010-06 | Faecal | France | 2010 |
| KT318086 | Caen 2008-04 | Faecal | France | 2008 |
| KT318085 | Caen 2007-03 | Faecal | France | 2003 |
| KT318083 | Caen 2005-02 | Faecal | France | 2005 |
| KT318084 | Caen 2005-01 | Nasal | France | 2005 |
| KT318087 | Caen 2003-05 | Faecal | France | 2003 |
| KF272913 | RVLC9 | Faecal | Ireland | 2011 |
| KF272911 | RVLC7 | Faecal | Ireland | 2011 |
| KF272909 | RVCL4 | Faecal | Ireland | 2011 |
| KF272915 | RVCL10 | Faecal | Ireland | 2011 |
| KM677156 | D72/11 | Nasal | Croatia | 2011 |
| KM677155 | D71/11 | Nasal | Croatia | 2011 |
| KM677147 | B27/10 | Faecal | Croatia | 2010 |
| GU808341 | WDBR-96 | Faecal | Brazil | 2010 |
| JF345157 | WDBR-B1 | Faecal | Brazil | 2013 |
| JF3451681 | USP-01 | Faecal | Brazil | 2012 |
| AY606193 | USP-03 | Faecal | Brazil | 2012 |

NI*= Not Identified
